# Global targetome analysis reveals critical role of miR-29a in pancreatic stellate cell mediated regulation of PDAC tumor microenvironment

**DOI:** 10.1186/s12885-020-07135-2

**Published:** 2020-07-13

**Authors:** Shatovisha Dey, Sheng Liu, Tricia D. Factora, Solaema Taleb, Primavera Riverahernandez, Lata Udari, Xiaoling Zhong, Jun Wan, Janaiah Kota

**Affiliations:** 1 Department of Medical and Molecular Genetics, Indiana University School of Medicine, Indianapolis, IN USA; 2 Department of Surgery, Indiana University School of Medicine, Indianapolis, IN USA; 3 The Melvin and Bren Simon Cancer Center, Indiana University School of Medicine, Indianapolis, IN USA

**Keywords:** Pancreatic cancer, PDAC, PSCs, microRNA, miR-29a, Protein interaction network, RNAseq, Desmoplasia, Tumor microenvironment, ECM

## Abstract

**Background:**

Pancreatic ductal adenocarcinoma (PDAC) is one of the most aggressive forms of malignancies with a nearly equal incidence and mortality rates in patients. Pancreatic stellate cells (PSCs) are critical players in PDAC microenvironment to promote the aggressiveness and pathogenesis of the disease. Dysregulation of microRNAs (miRNAs) have been shown to play a significant role in progression of PDAC. Earlier, we observed a PSC-specific downregulation of miR-29a in PDAC pancreas, however, the mechanism of action of the molecule in PSCs is still to be elucidated. The current study aims to clarify the regulation of miR-29a in PSCs and identifies functionally important downstream targets that contribute to tumorigenic activities during PDAC progression.

**Methods:**

In this study, using RNAseq approach, we performed transcriptome analysis of paired miR-29a overexpressing and control human PSCs (hPSCs). Enrichment analysis was performed with the identified differentially expressed genes (DEGs). miR-29a targets in the dataset were identified, which were utilized to create network interactions. Western blots were performed with the top miR-29a candidate targets in hPSCs transfected with miR-29a mimic or scramble control.

**Results:**

RNAseq analysis identified 202 differentially expressed genes, which included 19 downregulated direct miR-29a targets. Translational repression of eight key pro-tumorigenic and -fibrotic targets namely IGF-1, COL5A3, CLDN1, E2F7, MYBL2, ITGA6 and ADAMTS2 by miR-29a was observed in PSCs. Using pathway analysis, we find that miR-29a modulates effectors of IGF-1-p53 signaling in PSCs that may hinder carcinogenesis. We further observe a regulatory role of the molecule in pathways associated with PDAC ECM remodeling and tumor-stromal crosstalk, such as INS/IGF-1, RAS/MAPK, laminin interactions and collagen biosynthesis.

**Conclusions:**

Together, our study presents a comprehensive understanding of miR-29a regulation of PSCs, and identifies essential pathways associated with PSC-mediated PDAC pathogenesis. The findings suggest an anti-tumorigenic role of miR-29a in the context of PSC-cancer cell crosstalk and advocates for the potential of the molecule in PDAC targeted therapies.

## Background

Despite considerable advancement in the knowledge of pathogenesis and therapeutics of pancreatic ductal adenocarcinoma (PDAC) in recent years, the disease continues to remain as one of the deadliest malignancies. PDAC ranks as the seventh leading cause of cancer-related deaths worldwide [1] and the fourth in the United States [2]. This rapidly metastatic cancer is characterized by abundant desmoplastic reactions around pancreatic tumors mediated by the pancreatic stellate cells (PSCs) [3–5]. PSCs remain in quiescent state in normal pancreas, with a low extracellular-matrix (ECM) producing capacity. During pancreatic injury or inflammation, PSCs are activated by pro-inflammatory cytokines and growth factors to differentiate into myofibroblasts, expressing alpha smooth muscle actin (α-SMA) [3, 6, 7]. The transformed and activated stromal PSCs interact with the tumor cells, proliferate and produce ECM proteins and growth factors promoting fibrosis, pancreatitis and pancreatic cancer [4, 8, 9].

MicroRNAs (miRNAs) are a class of small (~ 22 nucleotide long), non-coding RNAs in multicellular organisms, which modulate key cellular mechanisms of proliferation, metabolism and apoptosis via post-transcriptional regulation of hundreds of genes [10]. miRNAs are initially generated as primary transcripts (pri-miRNA) from inter- and intragenic chromosomal regions predominantly via RNA polymerase II mediated transcription, and are then further processed by the Drosha RNase III enzyme to produce short hairpin pre-miRNAs [11]. Pre-miRNAs are exported to the cytoplasm by exportin 5, where they are further processed by the exonuclease III enzyme Dicer, in a complex, to generate mature miRNA. Mature miRNA, along with Agonaute 2, forms an RNA-dependent silencing complex and binds to the 3′-UTRs of the target gene mRNAs with imperfect complementarity to cause their degradation or translational suppression [11, 12]. Accumulating evidences have shown the involvement of miRNAs in regulation of pathological processes of variety of diseases including oncogenesis [12–14]. Studies have further demonstrated the association of dysregulated miRNAs in stromal cells with progression of different types of cancer, including pancreatic cancer, indicating the potential of miRNAs in developing targeted therapies [15–20].

In our previous work, we found microRNA-29a (miR-29a) to be pre-dominantly an anti-fibrotic molecule in PDAC, where miR-29a was significantly downregulated in activated PSCs and fibroblasts of murine and human PDAC as compared to normal pancreas, resulting in enhanced stromal extracellular matrix (ECM) deposition in PDAC microenvironment [21]. In addition, co-culture of pancreatic cancer cells with miR-29a overexpressing PSCs resulted in significant reduction in colony formation ability of the cancer cells and stromal deposition [21]. Thus, given the anti-fibrotic and tumor suppressive role of miR-29a in PSC-mediated PDAC progression, in the current study, we sought to decipher the mechanism of miR-29a in PSC regulation by identifying some of the key downstream target genes of the molecule, which also have critical functional implications in stromal remodeling and PDAC pathogenesis. Here we show for the first time that miR-29a concatenates genes belonging to key pathways associated with PDAC microenvironment, indicating the importance of the molecule in PSC-mediated PDAC stromal accumulation, suggestive of the potential of miR-29a as a therapeutic target for normalization of PDAC stroma.

## Methods

### Cell culture

Primary human pancreatic stellate cells (hPSCs) (3830, ScienCell Research Laboratories Carlsbad, California) were cultured in Dulbecco’s Modified Eagle Medium (DMEM, 11965092, Life Technologies, Carlsbad, CA) supplemented with 10% FBS in a humidified 5% CO_2_ incubator at 37 °C. hPSCs were authenticated using short tandem repeat profiling, and were regularly tested for mycoplasma contamination (MycoAlert, Lonza). All cells used in this study were less than passage 9.

### Transfection

To overexpress miR-29a, hPSC cells were seeded at 1 X 10^5^ cells/well in 6 well-plates for 24 h and transfected with control (CN-001000-01, GE Dharmacon, Lafeyette, CO) or miR-29a mimic (C-300504-07, GE Dharmacon, Lafeyette, CO) using DharmaFECT 1 Reagent (T-2001-01, GE Dharmacon, Lafeyette, CO) following manufacturer’s instructions. Total protein or RNA was isolated 48 h post-transfection for western blot or qPCR analyses, respectively.

### RNA extraction

Total RNA from cultured cells were extracted using the RNeasy plus Mini kit (74,134, Qiagen, Venlo, Netherlands) following manufacturer’s protocol. The concentration and purity of the extracted RNAs were measured using a Nanodrop 2000 Spectrophotometer (Thermo Fisher Scientific, Carlsbad, CA).

### RNAseq

For RNAseq, the quality and integrity of the extracted RNA were evaluated by a Bioanalyzer 2100 (Agilent technologies, CA). Samples with RNA Integrity Number (RIN) > 7.0 were used for RNAseq. cDNA libraries were prepared using the TruSeq RNA library kit (Illumina Inc., San Diego, CA). The libraries were amplified and then sequenced on an Illumina Hiseq.2000 instrument (San Diego, CA) with 100 bp paired end reads per sample. The quality of the sequence data was analyzed using FastQC [22]. The reads were mapped to the human genome (hg38) using STAR (v.2.5) [23]. Uniquely mapped sequencing reads were assigned to genes based on Gencode 25 using featureCounts (v1.6.2) [24]. Genes with read count per million (CPM) < 0.5 in two or more samples were filtered out and gene expression profiles were normalized using trimmed mean of M values (TMM) method. Differentially expressed genes (DEGs) were assessed by cutoff *p*-value of less than 0.05 after false discovery rate (FDR) adjustment with amplitude of fold change (FC) of gene expression greater than 2 linear FC.

### Target prediction, functional enrichment and network analysis

Conserved miR-29a target genes were obtained using TargetScan (v7.1). The hypergeometric model was adopted to identify the overlap between DEGs and miR-29a predicted targets.

Functional enrichment analysis of the gene ontology (GO) terms and KEGG pathway analysis were performed using R package to investigate the biological functions and pathways of the identified genes. The protein-protein interaction networks of the genes were explored using the STRING database, version 11 [25].

### Quantitative real time PCR (qRT-PCR)

RNA was reverse transcribed to cDNA using High capacity cDNA Reverse Transcription kit (4368814, Thermo Fisher Scientific, Carlsbad, CA) with random primers for genes or custom primer pool for miRNA (Thermo Fisher Scientific, Carlsbad, CA). To measure mature miR-29a expressions, TaqMan qRT-PCR reactions were set up using TaqMan Fast Advanced Mastermix (4444557, Applied Biosystems Foster City, CA) with TaqMan probe and primers for mature miR29a (002112, Applied Biosystems, Foster City, CA) or U6 snRNA (001973, Applied Biosystems, Foster City, CA). To assay the mRNA levels of genes, qRT-PCRs were performed with PowerUp SYBR Green Mastermix (A25742, Applied Biosystems, Foster City, CA) and custom primers Table S1). miRNA and mRNA qRT-PCR were normalized to U6 and ACTB respectively. Samples were run in triplicates in a 10 μl final volume using ABI 7500 Real-Time PCR machine with standard settings. Relative expressions were analyzed using ΔΔCT method.

### Western blot

Protein lysates were prepared with RIPA Buffer (PI-89900, Thermo Fisher Scientific, Carlsbad, CA) and quantified using BCA Protein Assay Kit (23,225, Pierce Biotechnology, Waltham, CA). Equal amounts of total protein were loaded onto NuPAGE 4–12% Bis-Tris Gels (NP0323, Invitrogen, Carlsbad, CA). After electrophoresis, the gels were electrotransferred onto polyvinylidene fluoride membranes, blocked with 5% dry non-fat milk and incubated overnight at 4 °C with specific primary antibodies. The membranes were washed and then probed with corresponding HRP conjugated goat anti-mouse (31,430, Thermo Fisher Scientific, Carlsbad, CA) or goat anti-rabbit (31,460, Thermo Fisher Scientific, Carlsbad, CA) antibodies at 1:5000 dilution. To develop the blots, ECL detection kit (34,096, Thermo Fisher Scientific, Carlsbad, CA) was utilized and the images were captured on an Amersham Imager 600 (GE Healthcare, Chicago, IL). Densitometry analysis was performed using Image J software to quantify each protein band, which were then normalized against loading control GAPDH. The primary antibodies used in this study were anti-IGF-1 (ab9572, Abcam, Cambridge, MA), anti-COL5A3 (PA5–77257, Thermo Fisher Scientific, Carlsbad, CA), anti-E2F7 (ab56022, Abcam, Cambridge, MA), anti-MYBL2 (PA546845, Thermo Fisher Scientific, Carlsbad, CA), anti-ITGA6 (3750, Cell Signaling Technology, Danvers, MA), anti-CLDN1 (4933S, Cell Signaling Technology, Danvers, MA), anti-ADAMTS2 (3485, Cell Signaling Technology, Danvers, MA), and anti-GAPDH (MA5–15738, Thermo Fisher Scientific, Carlsbad, CA).

### Statistical analysis

All data were expressed as mean ± standard error of the mean (SEM) of three independent experiments. Statistical analysis was performed by ANOVA or Student’s t test. Statistical significance is indicated as *p < 0.05 or **p < 0.01 or ***p< 0.001**.**

## Results

### RNAseq and identification of DEGs

**Fig. 1:**
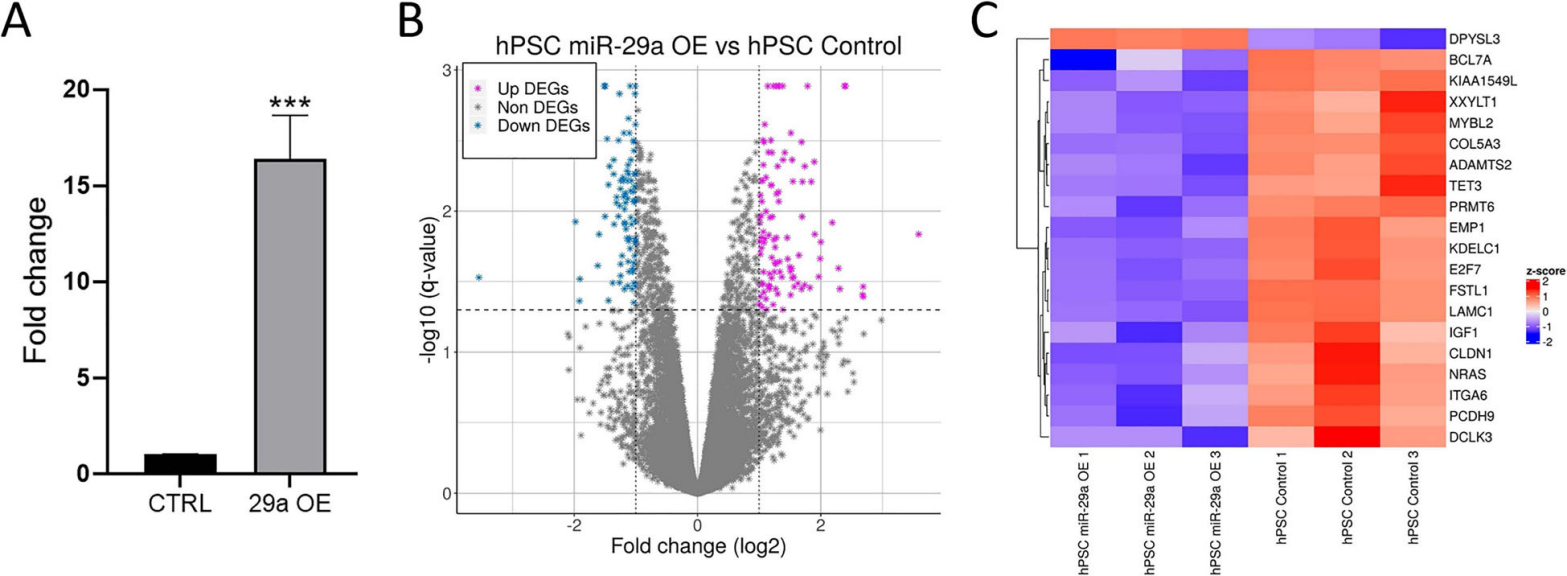
RNAseq analysis of miR-29a overexpressing hPSCs. **a** qPCR analysis for miR-29a expression in hPSCs transfected with miR-29a mimics (29a OE) as compared to hPSCs transfected with scramble control (CTRL). Numerical data are represented as average fold change (ΔΔCT) ± standard error of the mean (SEM); ***p < 0.001; *n* = 6. **b** Volcano plot of DEGs (log FC > 1 or < − 1, FDR < 0.05) in hPSC cells overexpressing miR-29a compared to controls. The horizontal axis represents log2 fold change between miR-29a overexpressing and control hPSCs. The negative log10 of the q-value is plotted on the vertical axis. Each point on the graph represents one gene. **c** A hierarchically clustered heatmap showing the expression patterns of the differentially expressed miR-29a direct target genes in the three replicates for each of miR-29a overexpressing (OE1, OE2 and OE3) and control (Control 1, Control 2, Control 3) mRNAs. Red and blue represent up- and downregulation respectively, and the color intensity represents the level of fold changes

RNAseq libraries were constructed using RNAs from control and miR-29a overexpressing hPSCs to generate global miR-29a targetome. Overexpression of miR-29a in the transfected hPSCs was verified by qPCR (Fig. 1a). Sequencing was performed with 2X 100 bp paired end reads. This yielded sequence reads ranging from 17 to 34 million pairs, of which 90–92% aligned to the hg19 genome assembly (Table 1). Quantile normalization with log_2_ transformation of number of counts per million (CPM) was performed and quality of raw sequencing reads and depth were verified for differential expression testing between the control and miR-29a overexpressing PSCs. For identification of DEGs, genes were plotted in a volcano plot by their log10 *P* values with FDR (q value) < 0.05 against log 2 fold change (FC) (Fig. 1b). This identified 90 downregulated and 106 upregulated genes with FDR < 0.05 and log FC < -1 or > + 1 respectively (Table S2). Next, inputting the DEG IDs into the TargetScan database, we identified 20 putative direct miR-29a targets among the identified DEGs- 19 of which were downregulated and one was upregulated (Fig. 1c). Among the downregulated miR-29a targets, IGF-1 exhibited the highest fold change, followed by COL5A3, E2F7, CLDN1, and MYBL2. DPYSL3 was the only upregulated target that met the screening criteria.

**Table 1:** RNA-Seq read counts and mapping statistics. Ctrl (Control) and miR-29a OE (overexpressing) represent hPSCs transfected with Control and miR-29a mimics respectively. R1, R2 and R3 are the three experimental replicates

Sample ID	Total Reads	Mapped Reads	Mapped High Quality Reads	Read Mapping Ratio	Percentage mapped to gene
**hPSC Ctrl R1**	38,372,969	35,691,075	35,333,674	92.07%	90.27
**hPSC Ctrl R2**	25,288,719	22,628,590	22,389,149	88.53%	90.09
**hPSC Ctrl R3**	18,759,093	16,958,204	16,698,047	89.01%	92.21
**hPSC miR-29a-OE R1**	36,899,783	34,288,605	33,965,913	92.05%	90.89
**hPSC miR-29a-OE R2**	20,971,993	18,594,180	18,318,771	87.35%	91.59
**hPSC miR-29a-OE R3**	33,726,399	29,480,256	29,128,167	86.37%	90.30

### GO term enrichment and pathway analysis of downregulated genes

GO analysis of the DEGs with an FDR < 0.05 revealed that the downregulated (target and non-target) genes were significantly enriched in several PDAC relevant biological processes such as regulation of mitosis and cell cycle, cell migration and motility, cellular adhesion, cell proliferation, extracellular matrix organization and cytokine signaling (Table 2). Among the 19 miR-29a predicted downregulated target genes, IGF-1, CLDN1 and ITGA6 were enriched in regulation of cell motility/migration (Table 2). COL5A3, ADAMTS2, ITGA6, LAMC1 and IGF-1 associated with mechanisms of ECM remodeling. While ITGA6 and IGF-1 are negative regulators of apoptosis, E2F7 and MYBL2 contribute to the regulation of cell cycle (Tables 2 and 3). In addition, the pathways enriched for miR-29a overexpressing PSCs included IGF-1 signaling, Tp53 signaling, collagen pathway, integrin-laminin interactions, RAS/MAPK signaling and cytokine signaling as depicted in Table 3. Thus, the GO and pathway enrichment analyses indicate that miR-29a modulates effectors of signaling pathways associated with crucial mechanisms of ECM remodeling and tumor-stromal crosstalk, suggesting a potential role of the molecule in PSC-mediated regulation of PDAC tumor microenvironment (TME).

**Table 2:** Most relevant biological processes associated with downregulated genes in miR-29a overexpressing hPSCs

Biological Process	Gene Name	Ratio	***p*** value
Positive regulation of cell proliferation	IGF1^a^; KIF14; IL1B; ESM 1; BCL2; KIF20B	6/490	0.024382
Cell division	CENPF; SPDL1; KIF14; SKA3; KIFC1; NEK2; SKA1; KIF18B; CENPE; CDCA5; KIF20B	11/346	4.69E-07
Regulation of G2/M transition of mitotic cell cycle	CENPF; KIF14; PLK4; NEK2; PLK1	5/80	3.2E-05
Negative regulator of extrinsic apoptotic pathway	ITGA6^a^; IGF1^a^	2/35	0.011085
Cell adhesion	LAMC1^a^; PCDH9^a^; PODXL; ITGA2; PCDH1; AJAP1	6/454	0.017486
Cell matrix adhesion	COL5A3^a^; ITGA6^a^; ADAMTS12; ITGA2	4/95	0.000932
Focal adhesion assembly	ITGA2; BCL2	2/27	0.006695
Positive regulation of fibroblast proliferation	IGF1^a^; E2F1	2/49	0.021029
Collagen fibril organization	COL5A3^a^; ADAMTS2^a^	2/46	0.018671
Extracellular matrix organization	COL5A3^a^; LAMC1^a^; ITGA6^a^; ITGA2; ABI3BP; PTX3	6/229	0.000624
Cell junction organization	ITGA6^a^;CLDN1^a^; LAMC1^a^; ITGA2	4/37	0.002963
Positive regulation of cell migration	CLDN1^a^; ITGA6^a^; IGF1^a^; PLAU; F2RL1; PODXL; LRRC15; IL1B	8/224	8.23E-06
Positive regulation of inflammatory response	ITGA2; IL1B	2/75	0.046007
Positive regulation of IL-6 secretion	F2RL1; IL1B	2/33	0.009895

^a^ miR-29a direct targets

**Table 3:** Pathways enriched for downregulated genes in miR-29a overexpressing hPSCs

Pathway name	Genes	***P*** value
Cell cycle	GINS1; PLK4; TOP2A; GINS2; BLM; CDCA5; PLK1; HJURP; CASC5; ESCO2; CENPA; AURKB; SKA1; CENPE; CENPF; EXO1; E2F1; E2F7^a^; NEK2; MYBL2^a^; SPDL1	*R* = 683; G = 21, *p* value = 2.07E-10
Tp53 pathway	BLM; EXO1; FANCD2; E2F1; SPDL1; E2F7^a^; AURKB	*R* = 259; G = 7; *p* value = 0.02074
Signaling by Ras mutants	NRAS^a^; IQGAP3	*R* = 54; G = 2; *p* value=6.41E-04
IGF pathway	NRAS^a^; LAMC1^a^; IGF1^a^; FSTL1^a^; PAPPA2	*R* = 127; G = 5; *p* value=0.032048
Laminin interactions	ITGA2; ITGA6^a^; LAMC1^a^	*R* = 31; G = 3; *p* value=0.003216579
Collagen binding	RC15; COL5A3^a^; ABI3BP; ITGA2; LRRC15	*R* = 53; G = 5; *p* value=0.00125
Collagen biosynthesis and metabolic pathway	COL5A3^a^; ADAMTS2^a;^ ITGA2	*R* = 84; G = 3; *p* value=0.04578

R = the number of reference genes in the category; G = number of genes in the gene set for each category; ^a^ miR-29a direct targets

### Validation analysis using qPCR and Western blots

**Fig. 2:**
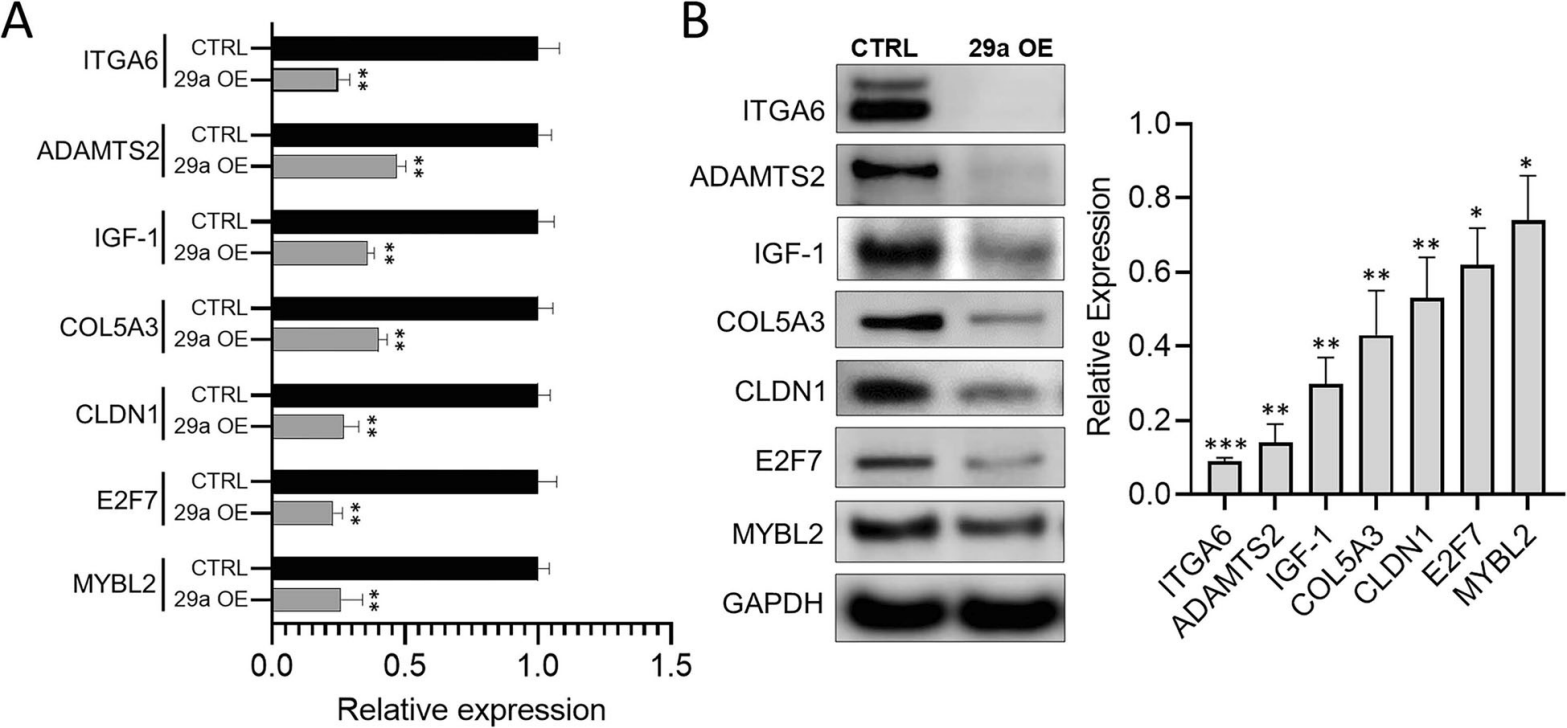
Validation of miR-29a direct target. **a** Relative fold changes estimated by qPCR analysis for the top miR-29a candidate target genes of ITGA6, ADAMTS2, IGF-1, COL5A3, CLDN1, E2F7 and MYBL2 in hPSCs transfected with miR-29a mimics (29a OE) compared with cells transfected with scramble control (CTRL). Numerical data are represented as average fold change (ΔΔCT) ± standard error of the mean (SEM); **p < 0.01; *n* = 3. **b** Total protein harvested from the hPSCs transfected with scramble control (CTRL) or miR-29a mimics (29a OE) 48 h post-transfection were subjected to western blot analysis for miR-29a candidate targets of ITGA6, ADAMTS2, IGF-1, COL5A3, CLDN1, E2F7 and MYBL2. GAPDH was used as the loading control. Quantification of band intensities normalized to GAPDH. Quantification of band intensities normalized to GAPDH and relative to respective controls are represented as ± SEM; *n* = 3, *p < 0.05, **p < 0.01, ***p< 0.001 (right). Uncropped blots are shown in Additional file 3: Fig. S1

Among the identified DEGs from the RNAseq, we selected all 19 down- and one upregulated miR-29a targets along with a subset of 24 additional DEGs to validate the RNAseq results using qRT-PCR. The expressions of 43 of the 44 tested genes well matched between the RNAseq and qPCR analyses (Table 4, Fig. 2a). Based on pathway analyses and available literature, IGF-1, COL5A3, CLDN1, E2F7, MYBL2, ITGA6 and ADAMTS2 were the most prominent miR-29a targets involved with one or more essential signaling mechanisms associated with TME regulation (Tables 2 and 3). Therefore, we next sought to find if miR-29a had a translational impact on these genes in PSCs. Our western blot analysis showed that protein levels of each of the seven selected targets were significantly diminished in miR-29a overexpressing PSCs (Fig. 2b). The most robust depletion was observed for ITGA6, followed by ADAMTS2 and IGF-1 respectively. All these three significantly downregulated target genes associate with ECM remodeling or fibrotic mechanisms. ITGA6 is a member of the integrin family that are heterodimer cell surface receptors comprising of α and β chains [26]. Alpha 6 containing integrins (α6/β4 and α6/β6) are the primary receptors for laminins, including laminin1 (LAMC1), a major ECM component [26]. Further, ECM in interaction with cellular integrins forms a scaffold, and plays essential role in cell proliferation, migration/invasion and survival [26]. ADAMTS2, belonging to the ADAM metallopeptidase with thrombospondin type 1 motif (ADAMTS) family, is responsible for processing of collagen type I, II, III and V precursors (pro-collagens) into mature collagen by excision of amino-propeptide, which is essential for generation of collagen monomers and assembly of mature collagen fibrils [27, 28]. Inhibition of ADAMTS2 has been shown to reduce stromal deposition and modulate TGF-β1 signaling [27, 29]. IGF-1 plays an essential role in fibrotic processes in different organs including pancreas, liver and lung [30–32]. Recent reports demonstrate the association of IGF-1 in PSCs to promote stromal accumulation and basal growth rate in PDAC [33], as well as miR-29a-mediated regulation of the gene [34]. Interestingly, each of the seven tested targets have been shown to exhibit pro-tumorigenic effects. Together, the observations suggest an anti-fibrotic and tumor suppressive function of miR-29a in PSC mediated PDAC pathogenesis.

**Table 4:** qPCR validation of differentially expressed genes

Gene Symbol	RNAseq			qRT-PCR
	logFC	*p* value	FDR	logFC
**Downregulated**				
IGF1^a^	−1.59	0.00	0.01	−1.48
COL5A3^a^	−1.50	8.80E-07	0.00	−1.32
CLDN1^a^	−1.49	0.00	0.01	−1.89
E2F7^a^	−1.49	1.21E-06	0.00	−2.12
MYBL2^a^	−1.35	1.42E-05	0.00	−1.92
TET3^a^	−1.24	3.92E-05	0.00	−1.13
PCDH9^a^	−1.2	3.02E-05	0.00	−1.18
EMP1^a^	−1.19	4.09E-06	0.00	− 2.18
ITGA6^a^	−1.18	0.00	0.012	−2.01
XXYLT1^a^	−1.13	7.18E-05	0.00	−1.08
BCL7A^a^	−1.12	0.00	0.02	−1.80
ADAMTS2^a^	−1.11	2.52E-05	0.00	−1.08
DCLK3^a^	−1.10	0.00	0.02	−1.32
LAMC1^a^	−1.09	9.16E-07	0.00	−1.32
KIAA1549L^a^	−1.07	1.43E-05	0.00	−1.12
PRMT6^a^	−1.07	3.82E-05	0.00	−1.19
KDELC1^a^	−1.03	6.87E-06	0.00	−1.83
NRAS^a^	−1.01	2.61E-05	0.00	−1.15
FSTL1^a^	−1.0	1.16E-06	0.00	−2.3
PPP1R14C	−3.54	0.00	0.02	−2.58
ESM 1	−1.97	0.00	0.01	−1.41
BCL2	−1.90	0.00	0.03	−1.30
PLAU	−1.51	4.93E-07	0.00	−2.17
IL1B	−1.26	9.42E-05	0.00	−2.88
EXO1	−1.22	0.00	0.02	−1.62
ITGA2	−1.10	4.23E-06	0.00	−2.17
IQGAP3	−1.09	0.00	0.01	−1.20
BLM	−1.06	0.00	0.01	−1.04
E2F1	−1.03	0.00	0.02	−1.20
AURKB	−1.02	5.01E-05	0.00	−1.92
**Upregulated**				
DPYSL3^a^	1.09	3.21E-06	0.00	1.01
PYGM	3.58	0.00	0.01	3.64
CXCL5	2.40	5.34E-07	0.00	1.98
NEFL	1.98	0.00	0.02	1.26
GNAO1	1.82	0.00	0.03	1.44
TNFRSF10C	1.62	0.00	0.03	1.20
HLA-DMA	1.51	0.00	0.03	2.06
ITGA7	1.46	0.00	0.02	1.65
FBXO32	1.41	1.06E-05	0.00	1.31
PIK3AP1	1.31	8.41E-05	0.00	1.53
HERC6	1.15	0.00	0.03	1.04
*HIST1H1C*	*1.12*	*0.00*	*0.04*	*−1.18*
IGFBP3	1.06	5.82E-06	0.00	0.89
HIST2H2BE	1.03	0.00	0.018	0.65

^a^miR-29a direct targets

### Network interactions of the downregulated miR-29a targets

**Fig. 3:**
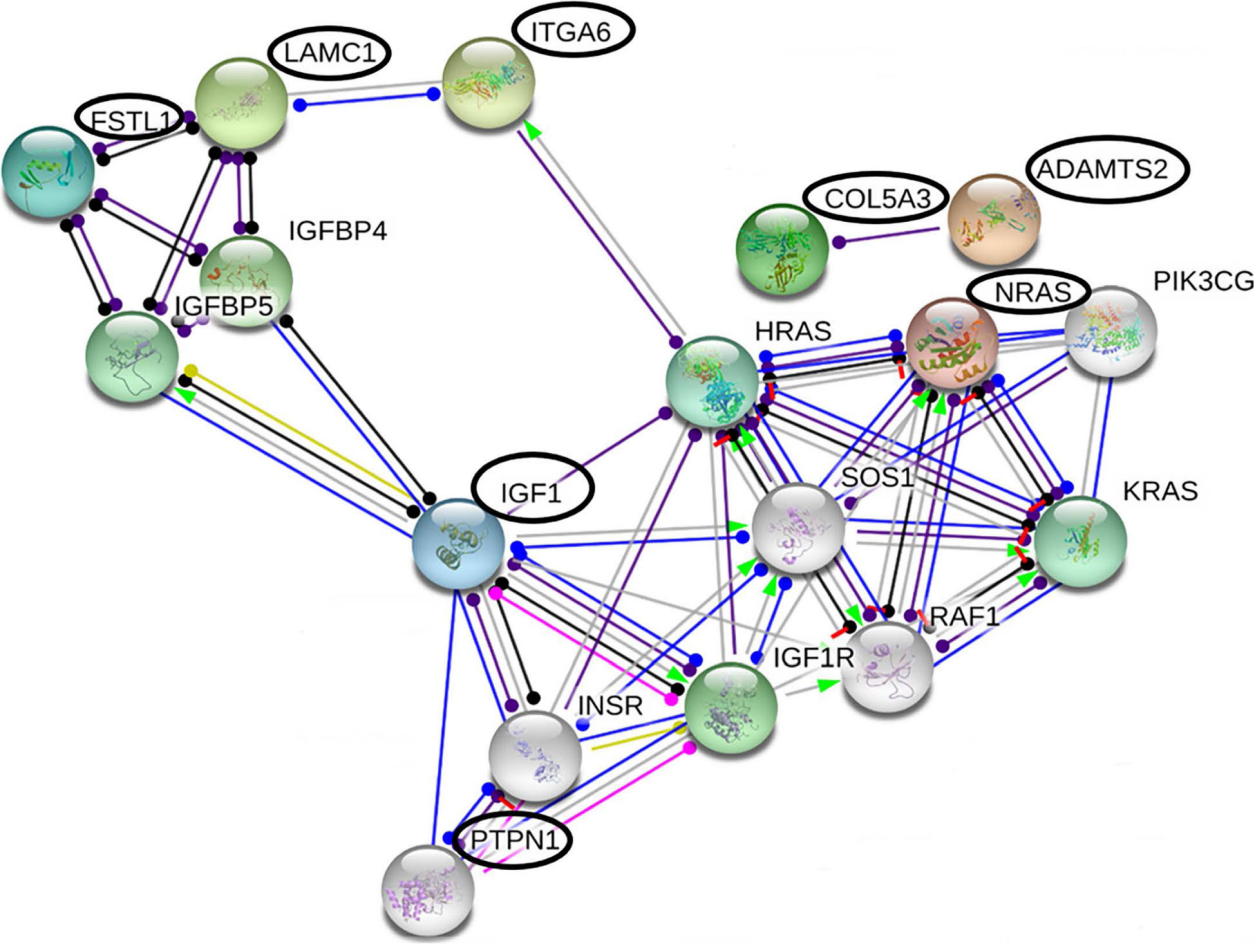
Network analysis for miR-29a predicted targets. Network interaction of miR-29a targets identified by RNAseq was constructed using the STRING database. The genes highlighted in black circles are the predicted miR-29a targets

To determine if the identified downregulated miR-29a direct target genes formed a network of interactions, we next analyzed the genes utilizing the Search Tool for the Retrieval of Interacting Genes/Proteins (STRING) database. We included a few additional nodes to construct the network. We observed three distinct networks in the interactome, which consisted of insulin/IGF, RAS/MAPK and laminin signaling pathways (Fig. 3).

IGF-1, belonging to the IGF family members, is one of the key regulators of the insulin/IGF pathway. IGF-1 is a direct downregulated miR-29a target in our dataset, which interacts with other effectors of the pathway including IGF-1R, INSR, IGFBP4 IGFBP5 and FSTL1 (Fig. 3). Interestingly, one of the oncogenes PTPN1 in the pathway is also a predicted direct miR-29a target, however, our RNAseq data did not show differential expression for this gene with miR-29a overexpression, which could be an effect specific to the PSCs. Nonetheless, the insulin/IGF signaling is a key driver in tumor-stromal interactions, metastasis and PDAC progression [33]. IGF-1 secreted by activated PSCs and fibroblasts in PDAC stroma via IGF-1 receptor (IGF-1R) promote cancer cell migration, invasion and metastasis [33, 35]. In fact, the RAS/MAPK pathway identified in our study consisted of interactions of IGF-1 and IGF-1R with other genes in the pathway including NRAS, HRAS, KRAS, SOS1 and RAF1. It is well documented that the MAPK signaling cascade bridges the crosstalk between ECM-mediated extracellular signaling through growth factors and their receptors such as IGF-1/IGF-1R, and subsequent intracellular response to allow cancer cell proliferation and migration [36]. IGF-1 bound activated IGF-1R phosphorylates insulin receptor substrates (such as IRS1, IRS2 and Shc). The Src homology 2 (SH2) domains of these substrates are recognized by signaling molecules to activate the intracellular effectors such as RAS, RAF and SOS and the RAS/MAPK pathway [37, 38]. Interestingly, in our previous study, we observed significant downregulation of NRAS with miR-29a overexpression in PDAC cell lines [39]. In the current study, miR-29a overexpression also resulted in moderate downregulation of NRAS in PSCs (logFC = − 1.01), however the role of NRAS in PSCs is unknown. Nonetheless, it is apparent that miR-29a modulates extracellular IGF-1/IGF-1R signaling in PSCs, and intracellular NRAS expression in pancreatic cancer cells, which indicates a functional role of the molecule in tumor-stromal crosstalk via insulin/IRF -RAS/MAPK signaling mechanism in PDAC.

The identified interactome further consisted of three miR-29a targets namely ITGA6, LAMC1 and FSTL1 that associate with laminin interactions, which are salient to pancreatic ECM and desmoplasia [40–42]. LAMC1 encodes for laminin γ1 chain isoform, which are essential non-collagenous ECM glycoproteins, integral to basement membrane assembly and crucial for intra- and extracellular communication to modulate cellular behavior [43]. Laminin interactions, including that of LAMC1, have been shown to promote oncogenesis via processes including cancer cell migration, differentiation and metastasis [44–47]. Cytoplasmic laminin expression correlates with poor patient prognosis in pancreatic cancer [48] and has been shown as one of the most efficient ECM proteins to promote cell adhesion-mediated drug resistance [49]. Further, ECM-integrin interactions are found to be crucial for adhesion-mediated drug and resistance to chemotherapy [50, 51].

## Discussion

In our previous studies, we observed significant loss of miR-29a in several PDAC cell lines [21, 39]. In addition, miR-29a was globally repressed in PDAC tumor tissues, as well as in a PSC- and epithelial cell- specific manner [21]. We further demonstrated that TGF-β1 via SMAD3 signaling negatively regulates miR-29a expression in PSCs and upregulates several ECM proteins including collagens, laminin and fibronectin [21]. In the current study, using RNAseq, we characterize the mechanism and pathway interactions by which miR-29a contributes to PSC-mediated regulation of ECM and tumor-stromal crosstalk. This will allow for a comprehensive understanding of the therapeutic applicability of the molecule in the context of PDAC stroma.

RNAseq analysis with miR-29a overexpressing PSCs and controls identified a number of DEGs, which included predicted direct and indirect targets of the molecule. Because miRNAs primarily regulate genes either by mRNA decay or translational repression, we focused on the direct targets that were downregulated with miR-29a overexpression. We validated the translational repression of the targets namely IGF-1, COL5A3, CLDN1, E2F7, MYBL2, which exhibited the highest fold changes in the RNAseq dataset, along with ITGA6 and ADAMTS2, which had functional relevance in stromal regulation. Our western blot analysis indicated the highest repression of ITGA6, ADAMTS2 and IGF-1 protein levels with miR-29a overexpression in PSCs (Fig. 2b). Among these identified direct targets, association of IGF-1 and COL5A3 with PSCs in PDAC has been reported previously [33, 52]. Network analysis with the targets identified three overlapping pathways related to IGF, RAS/MAPK signaling and laminin interactions. IGF-1 secreted by activated PSCs and CAFs via sonic hedgehog pathway activates IGF-1R in cancer cells triggering phosphorylation of insulin-receptor or Src substrates to promote PDAC metastasis via intracellular pathways such as RAS/MAPK [37, 53]. In addition, high IGF-1 with low IGFBP3 expressions associated with enhanced risks for PDAC [54]. Expectedly, patients with advance clinical stages (II and III) of PDAC had higher levels of IGF-1R and low IGFBP3, and exhibited poor prognosis [54]. Interestingly, the IGF-1R expressions in these patients associated with high stromal abundance, suggesting the regulation of tumor-stromal crosstalk via IGF/IGF-1R signaling [54]. Another identified miR-29a target CLDN1 is a tight junction protein that facilitates cell-ECM communication and EMT in various cancer types [55–57]. The gene is shown to be a contributor in tumor-stroma crosstalk in pancreatic cancer [58]. Although the regulation of CLDN1 in PSCs has not been reported previously, studies have shown the gene to be under the regulation of IGF-1 signaling [59, 60]. Upregulation of collagens, including COL5A3, is a salient feature of fibrosis and malignant tumor stroma, including that in PDAC [52, 61, 62]. Collagens are abundantly expressed in PDAC ECM; and collagen V, by binding with α2β1 integrin receptors, stimulates migration, proliferation and metastasis in PDAC [63]. Interestingly, ADAMTS2, another identified miR-29a downregulated target, primarily functions to process collagens I, II, III and V precursors into mature molecules [27, 28]. The gene promotes fibrosis via activation of TGF-β signaling [64]. Evidently, miR-29a plays an anti-fibrotic role in PDAC by influencing ECM deposition via modulation of multiple targets in the collagen pathway. In addition to these genes that directly regulate tumor-microenvironment and desmoplasia, the top targets identified from our dataset consisted of the two additional genes E2F7 and MYBL2, which play essential roles in cell cycle regulation. E2F7 associates with poor patient outcome in several types of cancer including PDAC [65–67] and has been shown essential for mouse embryonic survival [68]. Inhibition of E2F7 enhanced G1 phase percentage in prostate cancer reducing cellular proliferation [67]. Similarly, MYBL2 is a transcription factor which promotes cell proliferation and differentiation by fostering cell cycle entry into S and M phases, and is dysregulated in types of cancer [39, 69, 70]. A recent study demonstrated the regulatory role of MYBL2 in promoting PDAC desmoplasia and PSCs’ growth through sonic hedgehog and adrenomedullin via paracrine and autocrine signaling [71], however the role of the gene in PSCs has not been reported. A negative feedback regulatory mechanism between miR-29a and MYBL2 influencing the activation of PSCs is possible, but this requires future validation. Nonetheless, the identified set of miR-29a target genes exhibit a pro-fibrotic and tumorigenic function in PDAC desmoplasia and progression via multiple targeted pathways, although PSC-specific function of some of the identified target genes such as E2F7, CLDN1, MYBL2 and ADAMTS2 has not been studied previously. Together, the observations in the current study signify that overexpression of miR-29a may lead to inhibition of PSC-induced pro-fibrotic and desmoplastic effects by targeting these genes to impair signaling mechanisms such as sonic hedgehog, IGF, RAS/MAPK, collagen metabolism and laminin pathways, and perturbing their normal cellular responses to promote PDAC progression.

As mentioned above, IGF-1 signaling axis is a key mechanism that promotes PDAC tumor-stromal crosstalk and drug resistance. In our RNAseq dataset, we observed the most robust downregulation of the IGF-1 gene among all miR-29a targets. It is possible that in addition to IGF-1 alone, miR-29a regulates IGF-signaling via modulating multiple components of the pathway in PSCs, such as indirect regulation of genes including IGF-1R, INSR and direct targeting of some others. It is worthy to note that MYBL2 and E2F7 are miR-29a targets that are at the functional convergence of p53-IGF-1 pathways. Stromal p53 has been implicated as a key component that reprograms activated pancreatic and hepatic stellate cells to transform them into quiescent states [72, 73]. Depletion of p53 in stromal cells caused faster and more aggressive tumor development with enhanced invasion and metastasis of cancer cells, suggesting a paracrine mechanism of p53 in tumor progression [74, 75]. In addition, studies have reported the occurrence of inactivating p53 mutations in fibroblastic stromal cells and their association in promoting tumor progression and cancer cell metastasis in types of carcinogenesis [74], although the molecular mechanisms are still unclear. MYBL2 is a downstream effector of the p53 pathway [69]. With p53 mutations, MYBL2 repression is uncoupled allowing enhanced binding of the molecule with MuvB and FOXM1 leading to activation of mitotic genes [69, 76]. FOXM1 is an essential component of Akt signaling, which functions both in the context of tumor stroma and cancer cells to promote tumorigenesis [77–80]. Interestingly, Akt pathway is under inverse regulation of IGF-1 signaling [79, 81, 82]. Similarly, E2F7 is a crucial transcription factor, which promotes E2F1-p53 dependent apoptosis and cell-cycle arrest [68, 83]. In our RNAseq data with miR-29a overexpressing PSCs, we found E2F1 as one of the indirect downregulated targets. In addition, E2F7 has also been shown to be activated by Akt signaling in carcinomas [83–85]. Although the exact mechanisms of MYBL2 and E2F7 in PSCs is still to be understood, our results suggest that dysregulation of miR-29a in PSCs derepresses genes such as IGF-1, MYBL2 and E2F7, which may in turn disrupt stromal p53 regulation, promoting PSC-mediated tumor proliferation.

GO analysis showed that the direct and indirect miR-29a downregulated targets were enriched in crucial cellular and molecular functions associated with PDAC stromal remodeling and proliferation. The biological processes consisted of those related to cell cycle regulation, collagen formation, ECM organization and immune signaling (Table 2). Our study further identified inter-connected networks comprising of essential pathways in PDAC stromal regulation and desmoplasia (Table 3). Although a single miRNA is known to target hundreds of genes, resulting in their post- transcriptional repression, based on the functional network of the differentially expressed targets, the predominant phenotypic effect of a miRNA can be systematically analyzed in a context-specific manner. Our analysis using PSCs identifies a number of miR-29a target genes that are crucial players in PDAC stromal remodeling and tumor-stromal crosstalk, suggesting the importance of the molecule in their pathway regulations to modulate PDAC microenvironment and tumor progression.

## Conclusion

The current study is the first to use RNAseq platform for a comprehensive characterization of the PSC transcriptome under the regulation of miR-29a. In PDAC, activated PSCs foster cancer cell migration via desmoplastic reaction characterized by increased collagen, laminin and other ECM deposition resulting in fibrosis. Our data identified altered expressions of a number of novel genes under miR-29a regulation, including IGF-1, COL5A3, CLDN1, E2F7, MYBL2, ITGA6, ADAMTS2, and related pathways such as insulin-IRF, RAS/MAPK, laminin and collagen pathways in PSCs that are dysregulated or associate with PDAC tumor-stromal crosstalk and ECM remodeling. Given the functional relationship among the identified miR-29a targets in our PSCs dataset, it is likely that restoration of miR-29a in PSCs will dwindle or escalate the interconnected tumor-suppressive/pro-tumorigenic networks respectively in PDAC microenvironment, causing global regulation of the network functions to hinder the disease progression. Since our conclusions are primarily based on computational analysis, future investigations aimed to delineate the mechanistic relationship of miR-29a, its targets and related pathways in PSCs as well as cancer cells, would allow for a deeper comprehension of the associated pathological changes in tumor-stromal crosstalk in PDAC. This would be essential to assess the therapeutic modalities of miR-29a and its target networks in the disease. Nonetheless, our data in the current report identifies novel genes and networks under the regulation of miR-29a in PSCs, bolstering an anti-tumorigenic function of the molecule in the context of PDAC stroma. These findings suggest that targeted upregulation of miR-29a may hold great therapeutic value in efficacious PDAC treatment.

## Supplementary information

**Additional file 1: Table S1.** Primers for qPCR validation of differentially expressed genes in hPSCs.

**Additional file 2: Table S2.** Differentially expressed genes as identified by RNAseq analysis in miR-29a overexpressing hPSCs as compared to control cells.

**Additional File 3: Figure S1.** Full length blots of ITGA6, ADAMTS2, IGF-1, COL5A3, CLDN1, E2F7, MYBL2 and GAPDH in Fig. 2b. Red rectangles indicate cropped representative images presented in Fig. 2b.

## Data Availability

All sequence data have been deposited in the NCBI Gene Expression Omnibus (GEO) repository with the accession number GSE144767 or is available through *https://www.ncbi.nlm.nih.gov/geo/query/acc.cgi?acc=GSE144767**.*

## Abbreviations

ADAMTS2: ADAM Metallopeptidase With Thrombospondin Type 1 Motif 2
CLDN1: Claudin-1
COL5A3: Collagen alpha-3(V)
CPM: Counts per million
DEGs: Differentially expressed genes
E2F7: E2F Transcription Factor 7
ECM: Extracellular Matrix
EMT: Epithelial mesenchymal transition
FSTL1: Follistatin like 1
GO: Gene Ontology
IGF-1: Insulin-like growth factor 1
INS: Insulin
ITGA6: Integrin alpha-6
KEGG: Kyoto Encyclopedia of Genes and Genomes
LAMC1: Laminin subunit gamma 1
MAPK: Mitogen-activated protein kinase
miRNA: microRNA
MYBL2: MYB Proto-Oncogene like 2
PDAC: Pancreatic Ductal Adenocarcinoma
PSCs: Pancreatic stellate cells
qRT-PCR: Quantitative Real-time Polymerase Chain Reaction
STRING: Search Tool for the Retrieval of Interacting Genes/Proteins
UTR: Untranslated region
