# Supplementary material for: Global targetome analysis reveals critical role of miR-29a in pancreatic stellate cell mediated regulation of PDAC tumor microenvironment
Source: BMC Cancer. 2020 Jul 13;20:651. doi: 10.1186/s12885-020-07135-2 (PMC7359459; doi:10.1186/s12885-020-07135-2)
Supplement: Supplementary file 2 — Additional file 2: Table S2. Differentially expressed genes as identified by RNAseq analysis in miR-29a overexpressing hPSCs as compared to control cells. [file 12885_2020_7135_MOESM2_ESM.docx]

**Additional File 2: Table S2.** Primers for qPCR validation of differentially expressed genes in hPSCs.

| **Gene Name** | **Forward primer (5’-3’)** | **Reverse Primer (5’-3’)** |
| --- | --- | --- |
| IGF1 | TGTGGAGACAGGGGCTTTTA | ATCCACGATGCCTGTCTGA |
| COL5A3 | CCGACAAGAAGTTTGAGATCG | AGCCTCCAGGCTTTTCCTT |
| CLDN1 | CCTATGACCCCAGTCAATGC | ACAGCAAAGTAGGGCACCTC |
| E2F7 | CAGTTCAGGCTTCTGAGAGGA | CTAAAGAGTAGCCACCTGATCCTT |
| MYBL2 | GAGAAGTACGGACCCCTGAA | CCTCAGAACGCAGCACCT |
| TET3 | GTGCCTCCTTCTCCTTTGGT | CTCTTCCGGAGCACTTCTTC |
| PCDH9 | TGGAACAAATCATTCCTAACTGC | TCTCATCACTTATTTGGAGACAGC |
| EMP1 | TCACCATGGAGAAGGGAAAC | TAGATGGACACCCCCACAA |
| ITGA6 | AGCCTCTTCGGCTTCTCG | TTGGCTCTCTGCAGTGGAA |
| XXYLT1 | CTGGCTTCAAGTGCAAGGT | CTTCTGCATGGCCTCCAC |
| BCL7A | GAGGCCAAGGTGGATGAG | CGAATGTTCCATCGAGGACT |
| ADAMTS2 | CTAGCCGAAGCCTCCTCTGT | CTCCAAGGGTTCGATGAAGA |
| DCLK3 | CACGACAAGAGCATTGTCCA | ACCACATGCTTTGCAAGTCC |
| LAMC1 | CTGTTACTAGCCTCCTCAGCATTA | GCTTATTCAGGTCCACTGTATCC |
| KIAA1549L | TGCAGGCTACTTCCAGCTAAA | TCTGAGCAAACTTGCAGGAC |
| PRMT6 | ACTGAGCGTTGCCTTTTCTC | CCTACTTGCACGTGGGATCT |
| KDELC1 | CAGGCAGTGGATACATCAGG | ATTGCTCCTCTGGTGCTGAG |
| NRAS | GCAAGTCATTTGCGGATATTAAC | CATCCGAGTCTTTTACTCGCTTA |
| FSTL1 | GCCATCAATATTACAACGTATCCA | TCAATGAGAGCATCAACACAGA |
| PPP1R14C | TTACAGGAAGCTCTTGTAGACTGC | GCCTCTTATCCGAGAAAGCA |
| ESM1 | CATGGATGGCATGAAGTGTG | GGTGCCGTAGGGACAGTCT |
| BCL2 | AGTACCTGAACCGGCACCT | GCCGTACAGTTCCACAAAGG |
| PLAU | GGGCAGCACTGTGAAATAGATA | GCTGAAGGACAGTGGCAGA |
| IL1B | AAAGCTTGGTGATGTCTGGTC | GGACATGGAGAACACCACTTG |
| EXO1 | CTTTCTCAGTGCTCTAGTAAGGACTCT | TGGAGGTCTGGTCACTTTGA |
| ITGA2 | TCACAAGTGGGATTCAGTGC | CCATGAGATGTCTTCTGGACAA |
| IQGAP3 | TGAGGAGAGACTTTGCTGACTG | TTTTCCAGAAGCTCCACCAG |
| BLM | CGGATTTTGTTCCACCTTCTC | TTTCTGTCAGAGGTGTCAAGG |
| E2F1 | AAGTCCAAGAACCACATCCAGT | CTGGGTCAACCCCTCAAG |
| AURKB | GGAGAGTAGCAGTGCCTTGG | AGGCTCTTTCCGGAGGACT |
| DPYSL3 | TGTACTGAGCAGGCCAGAAG | TGCTCATGACCTTTGTGACG |
| PYGM | ACTTCTACGGCCATGTGGAG | ATGGCCAGTACCACCTGTGT |
| CXCL5 | GGTCCTTCGAGCTCCTTGT | GCAGCTCTCTCAACACAGCA |
| NEFL | ACCAAGACCTCCTCAACGTG | CACGCTGGTGAAACTGAGTC |
| GNAO1 | CCGCCAAAGACGTGAAATTA | CCGGAGAAGCCATCTTCAT |
| TNFRSF10C | CCCTAAAGTTCGTCGTCGTC | TGGTGGCAGAGTAAGCTAGGA |
| HLA-DMA | TAGGGCCAAAACTTGATGG | AGACCAAAGTGTTGGGCTTG |
| ITGA7 | AAGGAAAGCAAGGAGAACCAG | CACAAAGCAGCGACCAATC |
| FBXO32 | AAAGAGCGCCATGGATATTG | TGGAATCCAGAATGGCAGTT |
| PIK3AP1 | CGGCTGTGACGAGGATCT | CTGGCCTGAAGCATTTCAAC |
| HERC6 | ACCACTCCCTGGCATTATCA | CCAGGGACCTCACCCTCT |
| HIST1H1C | CCACTTGTACCCGAGTTTTTG | TCTTTACAGGGGCCTTCTCC |
| IGFBP3 | AACGCTAGTGCCGTCAGC | CGGTCTTCCTCCGACTCAC |
| HIST2H2BE | TGCTTGACTCCAAAGGCTCT | AACAAGCTCTTTTCTAGTGATTAGGTG |
