# Supplementary figures and images for: Global targetome analysis reveals critical role of miR-29a in pancreatic stellate cell mediated regulation of PDAC tumor microenvironment
Source: BMC Cancer. 2020 Jul 13;20:651. doi: 10.1186/s12885-020-07135-2 (PMC7359459; doi:10.1186/s12885-020-07135-2)

**Figure S1**

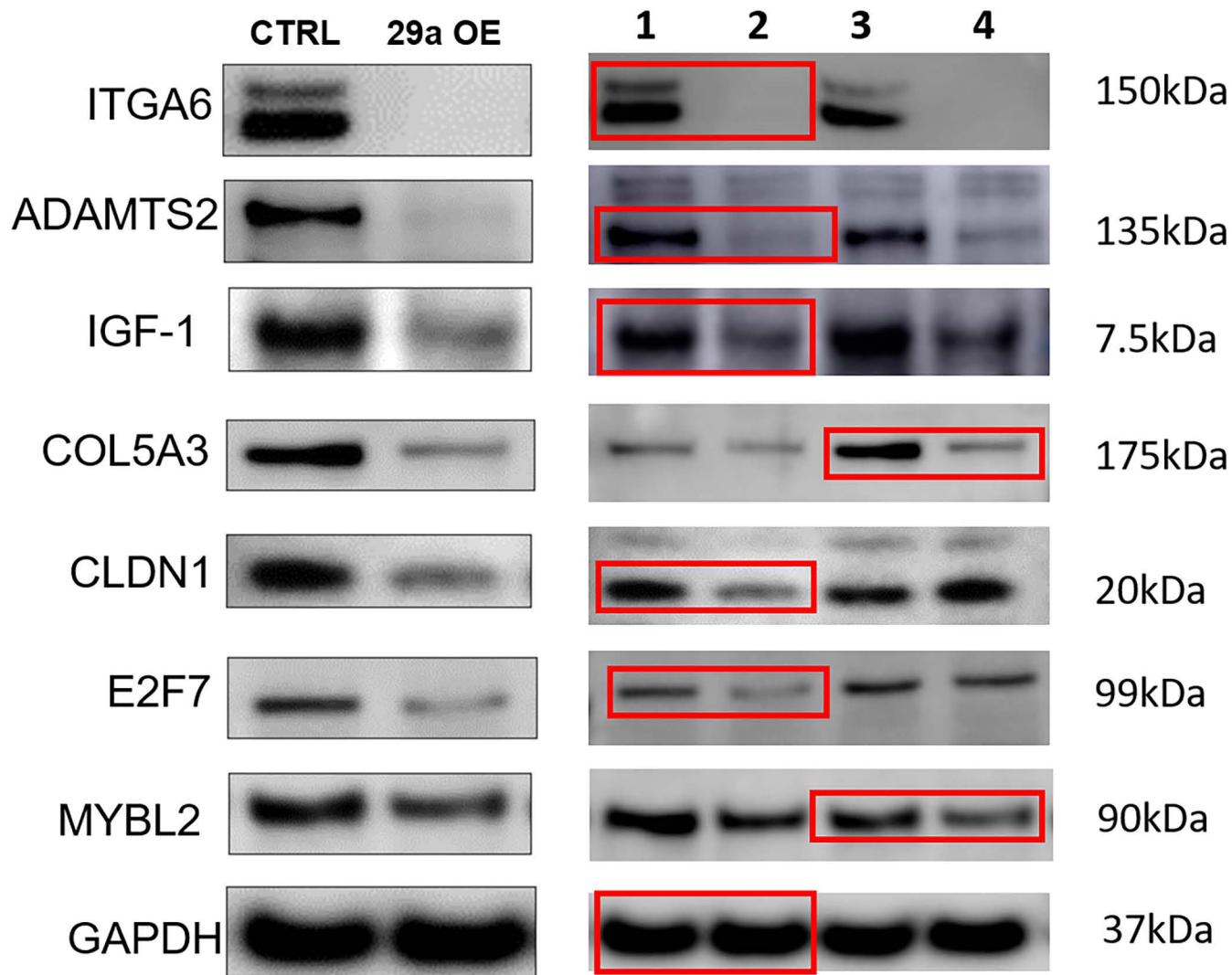

Supplement: Supplementary file 3 — Additional File 3: Figure S1. Full length blots of ITGA6, ADAMTS2, IGF-1, COL5A3, CLDN1, E2F7, MYBL2 and GAPDH in Fig. 2b. Red rectangles indicate cropped representative images presented in Fig. 2b. [file 12885_2020_7135_MOESM3_ESM.pdf]
